# Development of a Novel Nanotextured Titanium Implant. An Experimental Study in Rats

**DOI:** 10.3390/jcm8070954

**Published:** 2019-06-30

**Authors:** André Antonio Pelegrine, Peter Karyen Moy, Alireza Moshaverinia, Ana Lúcia do Amaral Escada, José Luis Calvo-Guirado, Ana Paula Rosifini Alves Claro

**Affiliations:** 1 Department of Research, Faculty of São Leopoldo Mandic, Campinas, Sao Paulo 13045-755, Brazil; 2 Department of Advanced Prosthodontics University of California, Los Angeles, CA 90095, USA; 3 School of Engineering, São Paulo State University, Guaratinguetá Campus, Sao Paulo 516-410, Brazil; 4 Department of Oral and Implant Surgery, Universidad Católica San Antonio de Murcia, 30107 Murcia, Spain

**Keywords:** osseointegration, osteogenesis, titanium, dental implants

## Abstract

This animal study evaluated the osseointegration level of a new nanotextured titanium surface produced by anodization. Ti-cp micro-implants (1.5 mm diameter by 2.5 mm in length) divided into two groups: titanium nanotextured surface treatment (Test Group) and acid etched surface treatment (Control Group). Surface characterization included morphology analysis using scanning electron microscopy and wettability by measuring contact angle. Sixteen Wistar rats were submitted to two micro implants surgical placement procedures. In each rat, one type of micro implant placed in each tibia. The animals sacrificed after two (T1) and six weeks (T2) post-implantation. After the euthanasia, tibias processed for histomorphometric analysis, which allowed the evaluation of bone to implant contact (BIC) and the bone area fraction occupancy between the threads (BAFO). Our surface analysis data showed that the Control Group exhibited an irregular and non-homogenous topography while the Test Group showed a nanotextured surface. The Test Group showed higher wettability (contact angle = 5.1 ± 0.7°) than the Control Group (contact angle = 75.5 ± 4.6°). Concerning the histomorphometric analysis results for T1, Control and Test groups showed BIC percentages of 41.3 ± 15.2% and 63.1 ± 8.7% (*p* < 0.05), respectively, and for BAFO, 28.7 ± 13.7% and 54.8 ± 7.5%, respectively (*p* < 0.05). For T2, the histomorphometric analysis for Control and Test groups showed BIC percentages of 51.2 ± 11.4% and 64.8 ± 7.4% (*p* < 0.05), respectively and for BAFO, 36.4 ± 10.3% and 57.9 ± 9.3% (*p* < 0.05), respectively. The findings of the current study confirmed that the novel nanotextured surface exhibited superior wettability, improved peri-implant bone formation, and expedited osseointegration.

## 1. Introduction

Titanium and its alloys are used in the biomedical area, especially as orthopedic and dental implants, because of their excellent biocompatibility, corrosion resistance, chemical stability in the physiological environment and elasticity modulus closer to that of bone when compared to other metals [1]. The surface of biomedical implants influences their interactions with bone cells and extracellular matrix, which will determine the osseointegration level [2].

Many of the excellent properties of titanium are attributed to the presence of titanium oxide on its surface layer, which increases the biological activity and corrosion resistance [3]. It is known that modifications of the surface morphology of a biomaterial that mimics the architecture of natural tissue tend to improve the cellular interactions and the promotion healthy tissue formation [4]. In this sense, different surface treatment methodologies have been used by the implant industry since the decade of the 1970s, such as titanium plasma spray [5], hydroxyapatite spray [6], anodization [7], sandblasting [8], acid-etching [9],sandblasting/acid etching [10] and laser ablation [11], producing, in most cases, microstructured and, occasionally, nanostructured surfaces. Nanostructured materials have different physical and chemical properties and have been proposed as an alternative in the development of new biomaterials. Recent work has shown that cells in the human body are predisposed to interact with nanostructured surfaces, such as nanoscale or nanoparticle-containing surfaces, and nanoscale materials interact with some proteins more effectively than conventional materials [12]. TiO_2_ nanotubes, besides natural production, have some essential characteristics, such as vertical alignment, high surface area, and controllable geometry [13]. Currently, the creation of nanotubes has received considerable attention in dentistry and orthopedics by increasing cell proliferation and differentiation when compared to conventional titanium surfaces (i.e., without nanotreatments), resulting in higher levels of osseointegration [14]. Lang et al. demonstrated that sandblasting/acid etched surfaces with higher hydrophilicity have greater bone-to-implant-contact (BIC) after 2 and 4 weeks after implantation in humans [15]. High surface wettability materials are also related to an anti-inflammatory microenvironment, which may improve the healing response to biomaterials [16]. As wettability was shown to be enhanced by nanosurfaces [17,18], micro-/nanostructured implant surfaces mimicking natural bone architecture are expected to have positive effects on osseointegration. In this sense, the realization of hybrid topographies, with conventional surface treatments (e.g., double acid etching and sandblasting/acid etching), which promote micro roughness, followed by anodizing treatment (e.g., nanotubes and TiO_2_ nanopores) seem to represent a promising future for the orthopedic and dental implant industry. Moreover, despite the fact that microsurfaces result in a better osseointegration level when compared with smooth surfaces [19], it’s known that microsurfaces can promote osteoblast differentiation but inhibit its proliferation. On the other hand, a nano topography provides for both osteoblast differentiation and proliferation [20].

To address the current limitations in the implant surface characteristics, our group has recently developed a new nanotextured implant surface based on acid double-etch technique followed by anodization to promote the growth of TiO_2_ nanotubes. We have modified the morphological nanostructure of the implant surface to improve the bioactivity of the materials to develop better osseointegration. In this animal study, the osseointegration level (i.e., bone to implant contact and bone area fraction occupancy) of our novel nanotextured titanium implant was evaluated using micro implants installed in Wistar rats’ tibia, which is commonly used for the bone to implant contact evaluation [21,22].

## 2. Experimental Section

### 2.1. Surface Modification

For surface analysis, grade IV Ti-cp discs with 10 mm diameter and thickness of 3 mm provided by IntraOss (Itaquecetuba, SP, Brazil). Discs were ground with silicon carbide paper of 1200 grit with Ra value of 0.15 μm, cleaned ultrasonically in acetone and ethanol (5 + 5 min), and dried in the air before performing surface treatments. Discs divided into two groups: Control Group and Test Group.

Control Group discs were submitted to double acid-etched surface treatment. In this treatment, samples provided to a biodegradable neutral detergent solution based on the citric acid solution for 2 h, followed by rinsing. Then, the first acid etching was performed with 30% nitric acid for 60 min, followed by rinsing and oven drying with absolute air filtration. Subsequently, immersion in 30% sulfuric acid was carried out for 120 min, followed by a new rinse. Finally, the last acid etching with 30% nitric acid was run for 60 min, followed by fresh rinse and oven drying with absolute air filtration. Every acid conditioning step were performed at 60 °C.

Test Group discs were submitted to double acid-etched surface treatment (same treatment of Control Group) followed by anodization to grow TiO_2_ nanotubes. For TiO_2_ nanotube formation, the anodic oxidation at room temperature was carried out with a constant potential of 30 V for 3 h in an electrolyte containing ethylene glycol, ammonium fluoride (1.0 g NH_4_F) and water. After the anodization, the implants washed with deionized water, air-dried and, to promote anatase phase formation, the samples were annealed in air at 450 °C for 1 h.

### 2.2. Surface Characterization

The surface morphology of the samples was characterized by scanning electron microscopy (SEM) using a Magellan 400L instrument (FEI, Hillsboro, OR, USA). The wettability was evaluated using the sessile drop method. A 2 microliter drop of distilled water was placed on three predetermined locations per disk, with three discs analyzed (*n* = 9, where each drop is *n* = 1), at room temperature. For each drops the right and left contact angles were measured, and a mean was obtained. The water contact angle of each drop was measured using the sessile drop method with a goniometer (DSA 100 model, Kruss Company, Ltd., Hamburg, Germany).

### 2.3. Animal Study

**Figure 1:**
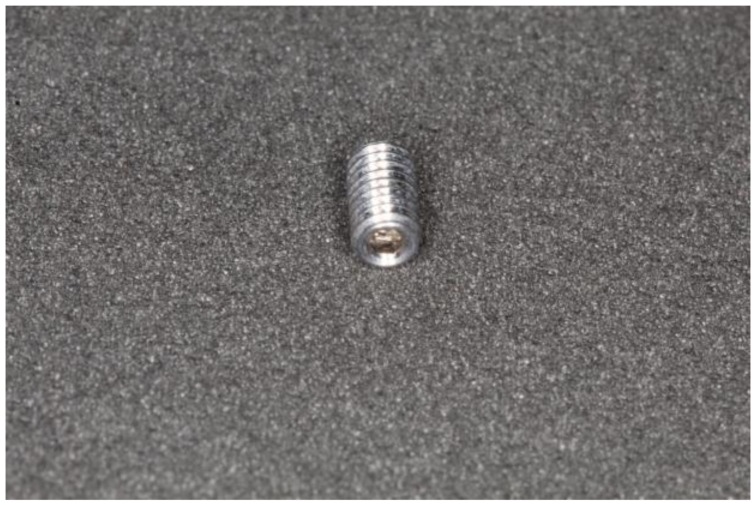
Grade IV Ti-cp micro implants with endosteal measurement of 1.5 mm in diameter and 2.5 mm in length.

The research project of the animal study was analyzed and approved by the ethics committee on animal research of Faculdade de Odontología e Centro de Estudos Odontológicos São Leopoldo Mandic, under the registration number 2018/001. Grade IV Ti-cp micro implants with endosteal measurement of 1.5 mm in diameter and 2.5 mm in length were produced by IntraOss (Figure 1).

Microimplants were divided into two groups following the same protocol used to discs. Then, micro implants of both groups were sterilized by gamma irradiation. Sixteen 12 weeks of age Wistar rats, weighing between 250 g and 300 g were used in this study. During the study period, the animals were kept in ventilated cages (two animals per cage) with light and dark cycles of 12 h, pelleted feed, and water ad libitum. Each rat received two implants, one per tibia (right side tibias received Control Group double etched micro-implants, and left side tibias received Test Group nanosurfaced micro-implants) using a www.randomization.com program. Eight rats were sacrificed after 2 weeks (T1), and eight rats were sacrificed in 6 weeks (T2), while eight animals were used in [23].

### 2.4. Surgical Technique 

The surgical procedure was done under general anesthesia induced by the inhalation of isoflurane (4.1% with 650 mL/min air flow) and maintained by continued administration of isoflurane (2.3% with 650 mL/min air flow). After trichotomy, the surgical area was decontaminated with povidone-iodine and local infiltration with 2% lidocaine + epinephrine 1:100,000 was subsequently administered. Then, an incision in the skin was done, at the tibia’s metaphysis region. After a full skin/periosteal flap was done to expose the tibia bone and a 1.2 mm diameter bur, adapted to the electric motor and rotating at 1000 rpm, was used to prepare the recipient bed with profuse irrigation with saline solution.

Thirty-two micro implants were placed, one per each tibia of each one of the sixteen animals. Each animal received one micro implant of each experimental group. Right side tibias received Control Group micro implants, and left side tibias received Test Group micro implants. The micro implants were manually installed with the aid of a 1.2 mm key until the micro-implant platform had contact with the cortical crest. After micro implants placement, the skin was sutured by layers, subcutaneously by using resorbable sutures (Vicryl 5-0; Johnson & Johnson Medical, New Brunswick, NJ, USA) and externally by using non-resorbable sutures (Mononylon 5–0; Ethicon, Johnson & Johnson Medical, New Brunswick, NJ, USA). All animals received subcutaneous injection of analgesic and were kept together in a cage in a temperature controlled room (18–20 °C). After 2 or 6 weeks the animals were sacrificed by anesthetic overdose. After removal of the skin and subcutaneous tissues, the tibias were removed and placed in a container with 10% formaldehyde for 5 days.

### 2.5. Histological Preparation and Histomorphometric Analysis

The samples were decalcified in 10% ethylenediaminetetraacetic acid for 4 to 8 weeks at room temperature. The 32 slides for histology (one per tibia) were made using a microtome to cut 7 μm sections of the entire set, including the titanium micro implant. The sections were stained with Stevenels’ blue and assessed under a light microscope. Digital images were obtained using a charge-coupled device digital camera (Rt Color, Diagnostic Instruments, Sterling Heights, MI, USA) attached to a light microscope (magnification ×1.25).

In order to create a specific image for each histological cut, Adobe Photoshop Elements 2.0 (Adobe Systems, San Jose, CA, USA) was used. A blind investigator traced all images using Image Pro Plus 4.5 Software for Windows (Media Cybernetics, San Diego, CA, USA). The following parameters were measured: 1) Bone to Implant Contact (BIC), which is the direct contact between bone and implant and 2) Bone Area Fraction Occupancy (BAFO), which is the area of mineralized tissue occupied between implant threads. These results were expressed as a percentage. A section of each tibia was analyzed in the central region of the implantation site.

### 2.6. Statistical Analysis

Commercially available software (GraphPad Prism 6.0 for Windows, GraphPad Software Inc., La Jolla, CA, USA) was used to compare all evaluated parameters and to create graphs. For the intergroup analysis, paired T-test was used. For the intragroup analysis, One-way ANOVA was used. A significance level of *p* < 0.05 was adopted. Mann-Whitney test for non-parametric statistics was performed for the analysis of BIC. The Kolmogorov-Smirnov test was used to assess whether the variables were normally distributed and Wilcoxon signed-rank test was performed to explore differences in osteogenesis around implants in both sides, with a significance level set at *p* < 0.005.

## 3. Results

**Figure 2:**
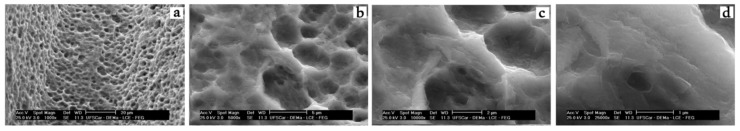
SEM images in scanning electron microscopy of Control Group. (**A**) 1000× magnification; (**B**) 5000× magnification; (**C**) 10,000× magnification; (**D**) 25,000× magnification.

**Figure 3:**
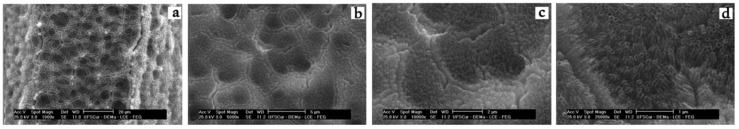
Image in scanning electron microscopy of Test Group. (**A**) 1000× magnification; (**B**) 5000× magnification; (**C**) 10,000× magnification; (**D**) 25,000× magnification.

In this study, we characterized the surface of our new nanotextured implant using SEM. Figure 2 shows the SEM images of the surface treated by double acid etched (Control Group) in low and higher magnification. A non-homogeneous and microporous layer was observed. On the other hand, the use of anodic oxidation after the double acid etching (Test Group) allowed the formation of relatively ordered TiO_2_ nanotubes, as observed in Figure 2 and Figure 3.

**Figure 4:**
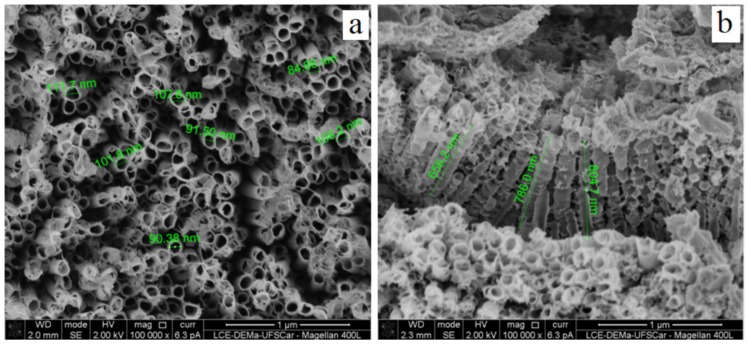
Image in scanning electron microscopy of test group. (**A**) 100,000× magnification showing nanotubes diameter; (**B**) 100,000× magnification showing the length of the nanotubes.

Further SEM analysis confirmed that the thickness of the TiO_2_ surface layer is within the nanometer range (Figure 4a,b).

**Figure 5:**
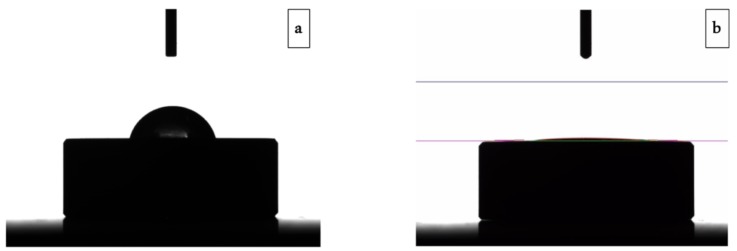
Wettability test of a control group sample (**A**) and test group sample (**B**). Note the difference in the contact angles between groups.

The wettability of the novel nanotextured titanium surface was also evaluated using contact angle measurements. Disc-shaped specimens were utilized in this study. The wettability test showed that TiO_2_ nanotubes (Test Group) promoted a more hydrophilic surface when compared with the standard double acid-etched surface (Control Group), which is demonstrated in Figure 5. Control and Test Group specimens had contact angles of 75.5 ± 4.6° and 5.1 ± 0.7°, respectively. Results are presented as mean ± standard deviation.

In the next step, Wistar rats were utilized for micro-implant surgical placement procedure and animals were sacrificed after two and six weeks of implant surgery. All animals were in good health conditions weighing between 300 g and 350 g at the time of the sacrifice. All animals used in this study (i.e., 16 Wistar rats) survived and were sacrificed after two (T1, *n* = 8) or six weeks (T2, *n* = 8) after implantation. In the histomorphometric analysis for T1, Control and Test groups showed BIC percentages of 41.3 ± 15.2% and 63.1 ± 8.7%, respectively, which was considered statistically significant (*p* = 0.0033).

For BAFO, the percentages were 28.7 ± 13.7% and 54.8 ± 7.5%, respectively, which was also considered statistically significant (*p* = 0.00032). For T2, the histomorphometric analysis for Control and Test groups showed BIC percentages of 51.2 ± 11.4% and 64.8 ± 7.4%, respectively, which was considered statistically significant (*p* = 0.0013). For BAFO, the portions were 36.4 ± 10.3% and 57.9 ± 9.3%, respectively, which was also considered statistically substantial (*p* = 0.0033). 

**Figure 6:**
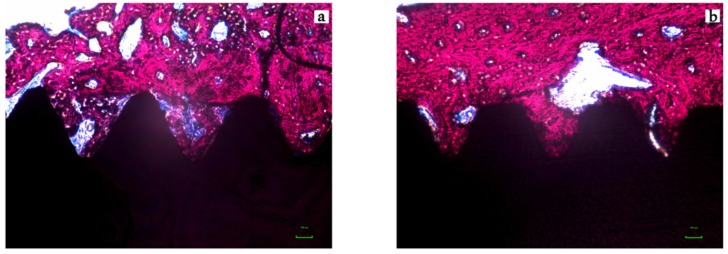
Photomicrograph of the micro-implants and surrounding tissues for the Control (**A**) and Test (**B**) groups in magnification of 200× (Stevenels’ Blue) for two weeks (T1). Bone mineralized tissue stained in pink, non-mineralized tissue stained in white/blue and titanium in black. Note that the level of bone to implant contact and the bone area occupied between the implants’ threads are higher in the test group.

**Figure 7:**
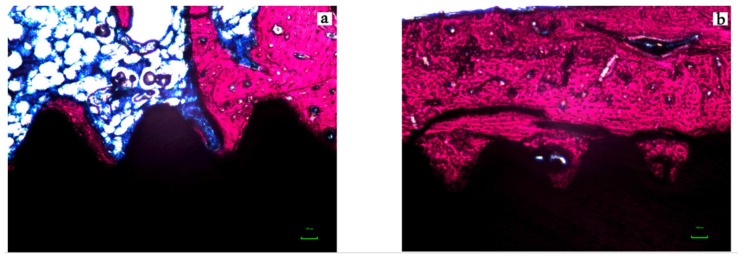
Photomicrograph of the micro-implants and surrounding tissues for the Control (**A**) and Test (**B**) groups in magnification of 200× (Stevenels’ Blue) for six weeks (T2). Bone mineralized tissue stained in pink, non-mineralized tissue stained in white/blue and titanium in black. Note that the level of bone to implant contact and the bone area occupied between the implants’ threads are higher in the test group.

**Figure 8:**
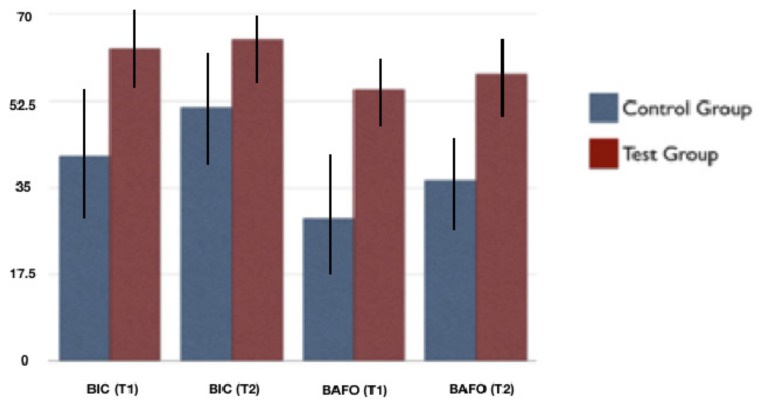
Comparison between groups (mean ± standard deviation, in %). BIC: bone-implant contact, BAFO: bone area fraction occupancy, T1: 2 weeks, T2: 6 weeks.

The representative histological images of each group can be seen in Figure 6a,b and Figure 7a,b and the histomorphometric results described above may be more closely visualized in Figure 8.

## 4. Discussion

The scientific literature has demonstrated that direct connection between bone and implant can be maximized through the production of micro-roughness on the surface of the implant (1–10 μm), which are traditionally produced by sandblasting, acid etching, spray plasma and other surface modification techniques [24]. Although micro-rough surfaces are likely to improve osseointegration, it is noteworthy that biomolecular interactions, protein adsorption and bone cell behavior are much more influenced by nanostructures (1–100 nm) than by microstructures (1–100 μm) [25,26]. In this context, nanoscale topography has been considered a promising strategy for the surface modification of titanium implants [27], and treatments that promote both micro and nano-structures on the surface of the implants seem to represent a trend. This justifies the creation of a micro-and nanostructured surface (hybrid topography) in the Test Group, where the double acid etching was performed (creating micro-roughness) followed by anodization (creating nanotubes of TiO_2_). This treatment increases the thickness of the titanium oxide layer, which has been related to an increase in biocompatibility and hemocompatibility [28]. The presence of surface nanostructures, when compared to traditional surfaces, increase the adsorption of the proteins, which favors the adhesion of osteoblasts [29].

The evaluation methods of the present study were in vitro and in vivo (animal study) assays. The in vitro tests were subdivided into surface morphology analysis (by Scanning Electron Microscopy—SEM) and wettability tests (by the water contact angle), made on titanium discs. The animal study had as primary and secondary outcomes the calculation of bone to implant contact (BIC) and bone area frequency occupancy (BAFO), respectively. The SEM analysis evidenced the presence of TiO_2_ nanotubes layer in the Test Group samples, differently than in the Control Group samples. The wettability tests showed a lower water contact angle in the examples of the Test Group 5.1 ± 0.7° when compared to the Control Group 75.5 ± 4.6°, which was considered statistically significant. As the samples of both groups were of the same titanium type (grade IV Ti-cp), coming from the same supplier, and having passed through the same method of sterilization, the only difference between them was the surface treatment. Thus, it can be stated that the drastic increase of the hydrophilicity achieved in the samples of the Test Group was due to the creation of the surface layer of TiO_2_ nanotubes, which certainly provided a significant increase of the specific surface area, as previously demonstrated by Wen et al. [30]. According to Lang and Chiang, the increased in hydrophilicity in titanium implants may have a positive repercussion in the acceleration of osseointegration, as verified by the histomorphometric results of the present study [15,31]. Hydrophilic surfaces maintain the conformation and function of proteins and, on the other hand, hydrophobic implant promote denaturation of proteins [32]. Moreover, as stated by Hotchkiss et al., high surface wettability materials are also related to an anti-inflammatory microenvironment, which may improve the healing response to biomaterials and, therefore, probably result in better osseointegration of implants [16].

Regarding the methods used in the animal study of the present study, there is a consensus in the scientific literature that bone-implant contact (BIC) and peri-implant bone level, widely reported in the literature as the bone area occupied between implants turns (BAFO), are not uniform. The quality of the osseointegration depends on the percentage of direct contact between the bone and the implant, as well as the preimplant bone density, the surface characteristics being a vital tool to improve the quality, especially in low-density bone tissue [33]. This justifies the accomplishment of the two types of histomorphometric measurements in the present study (i.e., BIC and BAFO). Pre-clinical studies have shown that surface treatments producing micro roughness compared to machined surfaces increase BIC and BAFO [34,35,36]. In this sense, the present study aimed to verify if the creation of a nanostructured surface on a previously treated surface with double acid etching could maximize and accelerate the process of osseointegration by calculating BIC and BAFO [37]. The option of using this evaluation methodology instead of a mechanical test, such as implant removal torque, or (ISQ) Implant Stability Quotient, is mainly due to two factors: (1) the analysis of BIC and BAFO allow real investigation of peri-implant bone tissue levels while removal torque and resonance frequency analysis only assess how much force is required to remove the implant and how stable the implant, respectively; and (2) the implant used in this study, because it is a tibial installation of a small animal, was only 1.5 mm in diameter, which made it impossible to make an internal connection that might allow the retriever and/or the installation of a transducer, which is imperative for stability measurement by resonance frequency analysis.

Histomorphometric results at two weeks after implantation (T1) demonstrated, with statistical significance, superiority of the Test Group in both evaluated parameters (i.e., BIC and BAFO). For BIC, the Test and Control Groups obtained 63.1 ± 8.7% and 41.3 ± 15.2%, respectively, which indicates an increase of almost 50% in the level of contact between bone and implant when using the surface with nanotubes. At the same time of evaluation (T1), for BAFO, the differences between Test Group and Control were even higher, with percentages of 54.8 ± 7.5% and 28.7 ± 13.7%, respectively. For the evaluation after 6 weeks of implantation (T2), BIC results for the Test and Control Groups were 64.8 ± 7.4% and 51.2 ± 11.4%, respectively. For BAFO, the percentages were 57.9 ± 9.3% and 36.4 ± 10.3%. The intergroup statistical comparisons showed that BIC and BAFO scores were higher in the Test Group, for both evaluation times. As the only difference between the micro-implants used in the Test and Control Groups was the presence or absence of surface TiO_2_ nanotubes, it can be inferred that this was the cause of the best histomorphometric results of the Test Group. The intra-group statistical comparisons showed a statistically significant difference for the BIC and BAFO parameters only for the Control Group, that is, there was a significant increase in the results between T1 and T2 only for the Control Group. The lack of increase in BIC and BAFO results for the Test Group between T1 and T2 is most probably because osteoconduction was superior and, consequently, osseointegration was earlier achieved when using a more hydrophilic surface, as is the case of the surface with the presence of TiO_2_ nanotubes.

It is known that microsurfaces can promote osteoblast differentiation but inhibit its proliferation. Therefore, even though microsurfaces result in a better osseointegration level when compared with smooth surfaces, the lack of osteoblast proliferation results in a smaller accumulation of bone mass compared with that yielded by a smooth surface [19,38], so it could explain the higher difference for BAFO than for BIC between Control and Test Groups. Therefore, the use of TiO_2_ nanotubes, probably by promoting both osteoblast differentiation and proliferation, optimized the accumulation of bone mass around the implant (e.g., BAFO) and, consequently, optimized the osseointegration. A recent study in dogs conducted by Lee et al., 2019 compared the bonding between the nanotube and machined titanium implant [39]. After 4 and 12 weeks the implants were removed with torque force and analyzed by SEM. The machined implants were partially covered with a bone while implants containing nanotubes were completely covered with bone, without any deformities in nanotubes. These results show that nanotubes have high bonding stability with titanium implants besides having a high level of osseointegration.

The animal used in the present study (i.e., the rat) is an attractive experimental model because bone turnover in rats is several times faster than in a human [40]. In this model, preliminary signs of bone formation appear about 1 week after implantation and complete bone formation achieved about 4 weeks [41,42]. Thus, in this study, euthanasia was chosen at 2 and 6 weeks, thus theoretically allowing an evaluation during the period of bone healing/osseointegration (T1) and another evaluation after bone remodeling/maturation period (T2). Therefore, from the translational point of view, the T1 results presented by the Test Group of the present study allow us to conjecture that the nanotubular surface affects the acceleration of osseointegration and the consequences of T2 that the nanotubular surface has repercussions on the maximization of osseointegration. These results allow us to envisage very favorable clinical implications (e.g., decreased treatment time and improved osseointegration) if confirmed by human studies. However, it’s important to state that, since this is an animal study, extrapolation to the clinic should be done with caution since biological behavior between rats and humans are different. Moreover, as osseointegration is achieved at a very high rate in a clinical setting [43], it’s also important to state that the main problem of dental implants is the onset of peri-implant inflammation. Thus, in this issue, the development of antibacterial surfaces developed by nanotechnology might improve the clinical outcomes and, therefore, should be investigated.

Advances in electrochemical anodization have led to the possibility of manufacturing self-ordered TiO_2_ nanotubes/nanopores on the titanium surface. This allowed, in the present study, the use of titanium implants with well-oriented nanotubes and a diameter of approximately 100 nm (see Figure 3). Nanotubes with a diameter around 100 nm have been related to an increase in alkaline phosphatase activity when compared to nanotubular surfaces of 30 to 70 nm [44]. As alkaline phosphatase is a marker of osteogenic differentiation, nanotubular surfaces with a diameter around 100 nm demonstrate integrative properties of bone tissue which probably had repercussion on the superior results in favor of the Test Group of the present study. Besides the benefits in the osseointegration aspect provided by the surface with TiO_2_ nanotubes, which can be observed by the results of the present study and corroborated by Wang et al., and Yu et al., [45,46], these nanostructures appear to have the potential to enable local release of drugs—such as antibiotics, proteins, growth factors, anti-inflammatories, etc.—due to the geometry of nanotubes (which resemble tiny open test tubes on the top, closed at the bottom) [47]. This feature opens up a wide range of possibilities and supports the use of nanotubes as a versatile strategy in the field of Implantology.

## 5. Conclusions

The findings of this study suggest that nanotextured surfaces with TiO_2_ nanotubes, created by anodization, improved the wettability of the titanium surface and also enhanced the peri-implant bone level, with an expedited osseointegration level when compared to a standard titanium surface treated by double acid etched.
